# Bis(2-thien­yl)acetyl­ene

**DOI:** 10.1107/S1600536809036812

**Published:** 2009-09-19

**Authors:** Emily M. Harcourt, Daniel E. Lynch, Darren G. Hamilton

**Affiliations:** a Department of Chemistry, Mount Holyoke College, South Hadley, MA 01075, USA; b Exilica Limited, The Technocentre, Puma Way, Coventry CV1 2TT, UK

## Abstract

The planar [maximum deviation 0.0066 (4) Å] symmetrical mol­ecule of the title compound, C_10_H_6_S_2_, lies across a crystallographic inversion centre. The thio­phene rings are rotationally disordered about the acetyl­ene bond, with the two pseudo inversion-related S atoms in 0.80:0.20 occupancy sites. The C C bond distance is 1.195 (9) Å.

## Related literature

For the preparation of the title compound, related diaryl­acetyl­enes and cobalt-containing metallocenes derived from these materials, see: Harrison *et al.* (1997 ▶); Harcourt *et al.* (2008 ▶). For recent synthetic organic uses, see: Yu & Rovis (2006 ▶); Geyer *et al.* (2008 ▶). The metal center employed in an acetyl­ene cyclo­oligomerization may also remain as an integral component of the product, or products, see: Rausch & Genetti (1970 ▶). For spectroscopic data, see: Mio *et al.* (2002 ▶).
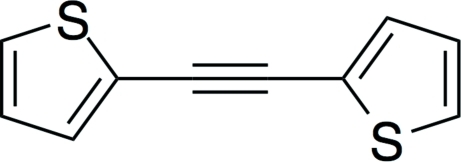


## Experimental

### 

#### Crystal data

C_10_H_6_S_2_
*M* *_r_* = 190.29
Orthorhombic, 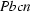

*a* = 10.6325 (15) Å
*b* = 10.8713 (15) Å
*c* = 7.5600 (5) Å
*V* = 873.85 (18) Å^3^
*Z* = 4
Mo *K*α radiation
μ = 0.54 mm^−1^
*T* = 120 K
0.55 × 0.05 × 0.03 mm

#### Data collection

Nonius KappaCCD diffractometer
Absorption correction: multi-scan (*SADABS*; Sheldrick, 2003 ▶) *T*_min_ = 0.755, *T*_max_ = 0.984
3812 measured reflections
849 independent reflections
493 reflections with *I* > 2σ(*I*)
*R*_int_ = 0.129

#### Refinement

*R*[*F* ^2^ > 2σ(*F* ^2^)] = 0.073
*wR*(*F* ^2^) = 0.173
*S* = 1.08
849 reflections
58 parameters
H-atom parameters constrained
Δρ_max_ = 0.41 e Å^−3^
Δρ_min_ = −0.41 e Å^−3^

### 

Data collection: *COLLECT* (Nonius, 1998 ▶); cell refinement: *DENZO* (Otwinowski & Minor, 1997 ▶) and *COLLECT*; data reduction: *DENZO* and *COLLECT*; program(s) used to solve structure: *SHELXS97* (Sheldrick, 2008 ▶); program(s) used to refine structure: *SHELXL97* (Sheldrick, 2008 ▶); molecular graphics: *PLATON* (Spek, 2009 ▶); software used to prepare material for publication: *SHELXL97*.

## Supplementary Material

Crystal structure: contains datablocks I, global. DOI: 10.1107/S1600536809036812/zs2008sup1.cif

Structure factors: contains datablocks I. DOI: 10.1107/S1600536809036812/zs2008Isup2.hkl

Additional supplementary materials:  crystallographic information; 3D view; checkCIF report

## Figures and Tables

**Fig. 1.**
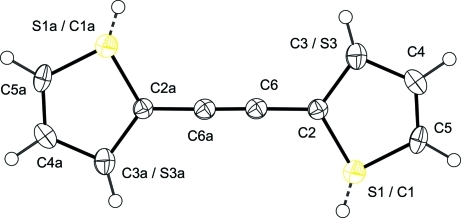
Molecular configuration and atom-numbering scheme for (I) showing inversion symmetry [symmetry code: (a) -x, -y + 1, -z]. Rotationally disordered thiophene S/C atom pairs are S1, C3 (S.O.F. 0.80) and S3, C1 (S.O.F. 0.20). Displacement ellipsoids are drawn at the 50% probability level.

## supplementary crystallographic information

### Comment

Diarylacetylenes are versatile components of metal-mediated cycloaddition reactions. Their relative ease of preparation from palladium catalyzed coupling of aryl iodides to acetylene has ensured their continued use in the development of new synthetic routes, for example, nitrogen containing heterocycles (Yu & Rovis, 2006), and new catalytic reaction methodologies such as alkyne–nitrile cross metathesis (Geyer *et al.*, 2008). The metal center employed in an acetylene cyclooligomerization may also remain as an integral component of the product, or products, as described in the seminal work of Rausch & Genetti (1970). The title compound bis(2-thienyl)acetylene (I) is found to have inversion symmetry coincident with crystallographic symmetry (Fig. 1). However, the two 2-thiophene residues are rotationally disordered about the acetylene bond with the two pseudo-inversion related S atoms having 80/20% occupancy. The C—C triple bond distance is 1.195 (9) Å.

### Experimental

The title compound was prepared by Sonogashira coupling of two equivalents of 2-iodothiophene to acetylene under standard conditions (Harrison *et al.*, 1997). Full experimental details (Harcourt *et al.*, 2008) and spectroscopic data (Mio *et al.*, 2002) have been previously published.

### Refinement

All H atoms were included in the refinement at calculated positions, in the riding-model approximation, with C—H distances of 0.95 Å. The isotropic displacement parameters for all H atoms were set equal to 1.25*U*_eq_ of the carrier atom. The refined site occupancy factors for the disordered atoms (S1, C3, H3) and (S3, C1, H1) of the pseudo-centrosymmetrically related thiophene rings were 0.80 (1), and 0.20 (1) respectively. Structure factor file checks indicate that there is only one listed reflection that is likely to have been affected by the beamstop.

### Crystal data

**Table:**

C_10_H_6_S_2_	*F*(000) = 392
*M**_r_* = 190.29	*D*_x_ = 1.446 Mg m^−^^3^
Orthorhombic, *P**b**c**n*	Mo *K*α radiation, λ = 0.71073 Å
Hall symbol: -P 2n 2ab	Cell parameters from 1041 reflections
*a* = 10.6325 (15) Å	θ = 1.0–27.5°
*b* = 10.8713 (15) Å	µ = 0.54 mm^−^^1^
*c* = 7.5600 (5) Å	*T* = 120 K
*V* = 873.85 (18) Å^3^	Needle, colourless
*Z* = 4	0.55 × 0.05 × 0.03 mm

### Data collection

**Table:**

Nonius KappaCCD diffractometer	849 independent reflections
Radiation source: Bruker Nonius FR591 rotating anode	493 reflections with *I* > 2σ(*I*)
10 cm confocal mirrors	*R*_int_ = 0.129
Detector resolution: 9.091 pixels mm^-1^	θ_max_ = 26.0°, θ_min_ = 2.7°
φ and ω scans	*h* = −13→11
Absorption correction: multi-scan (*SADABS*; Sheldrick, 2003)	*k* = −13→12
*T*_min_ = 0.755, *T*_max_ = 0.984	*l* = −9→8
3812 measured reflections	

### Refinement

**Table:**

Refinement on *F*^2^	Primary atom site location: structure-invariant direct methods
Least-squares matrix: full	Secondary atom site location: difference Fourier map
*R*[*F*^2^ > 2σ(*F*^2^)] = 0.073	Hydrogen site location: inferred from neighbouring sites
*wR*(*F*^2^) = 0.173	H-atom parameters constrained
*S* = 1.08	*w* = 1/[σ^2^(*F*_o_^2^) + (0.050*P*)^2^ + 3.1085*P*] where *P* = (*F*_o_^2^ + 2*F*_c_^2^)/3
849 reflections	(Δ/σ)_max_ < 0.001
58 parameters	Δρ_max_ = 0.41 e Å^−^^3^
0 restraints	Δρ_min_ = −0.41 e Å^−^^3^

### Special details

**Table:**

Experimental. The minimum and maximum absorption values stated above are those calculated in *SHELXL97* from the given crystal dimensions. The ratio of minimum to maximum apparent transmission was determined experimentally as 0.675726.

### Fractional atomic coordinates and isotropic or equivalent isotropic displacement parameters (Å^2^)

**Table:**

	*x*	*y*	*z*	*U*_iso_*/*U*_eq_	Occ. (<1)
S1	0.17278 (13)	0.28773 (15)	0.17139 (19)	0.0303 (6)	0.80
C1	0.17278 (13)	0.28773 (15)	0.17139 (19)	0.0303 (6)	0.20
H1	0.1105	0.2380	0.2263	0.038*	0.20
C2	0.1671 (4)	0.4188 (5)	0.0513 (6)	0.0220 (12)	
C3	0.2949 (3)	0.4642 (3)	−0.0126 (4)	0.0291 (9)	0.80
H3	0.3142	0.5342	−0.0827	0.036*	0.80
S3	0.2949 (3)	0.4642 (3)	−0.0126 (4)	0.0291 (9)	0.20
C4	0.3820 (5)	0.3703 (5)	0.0635 (6)	0.0295 (15)	
H4	0.4705	0.3749	0.0468	0.037*	
C5	0.3285 (4)	0.2786 (5)	0.1572 (6)	0.0271 (13)	
H5	0.3757	0.2144	0.2105	0.034*	
C6	0.0498 (4)	0.4768 (5)	0.0149 (6)	0.0235 (14)	

### Atomic displacement parameters (Å^2^)

**Table:**

	*U*^11^	*U*^22^	*U*^33^	*U*^12^	*U*^13^	*U*^23^
S1	0.0283 (8)	0.0314 (11)	0.0311 (8)	0.0002 (8)	−0.0017 (6)	0.0030 (7)
C1	0.0283 (8)	0.0314 (11)	0.0311 (8)	0.0002 (8)	−0.0017 (6)	0.0030 (7)
C2	0.022 (2)	0.023 (3)	0.021 (2)	−0.001 (2)	−0.0019 (19)	0.000 (2)
C3	0.0264 (16)	0.029 (2)	0.0322 (16)	0.0028 (17)	−0.0049 (13)	−0.0064 (15)
S3	0.0264 (16)	0.029 (2)	0.0322 (16)	0.0028 (17)	−0.0049 (13)	−0.0064 (15)
C4	0.021 (2)	0.035 (4)	0.032 (3)	−0.002 (3)	0.003 (2)	−0.008 (3)
C5	0.030 (3)	0.027 (3)	0.025 (2)	0.012 (3)	−0.007 (2)	−0.006 (2)
C6	0.025 (2)	0.021 (4)	0.025 (2)	−0.002 (2)	−0.001 (2)	−0.001 (2)

### Geometric parameters (Å, °)

**Table:**

S1—C2	1.691 (5)	C4—C5	1.349 (7)
C2—C6	1.424 (6)	C4—H4	0.95
C2—C3	1.525 (5)	C5—H5	0.95
C3—C4	1.493 (6)	C6—C6^i^	1.195 (9)
C3—H3	0.95		
			
C6—C2—C3	125.2 (4)	C5—C4—C3	116.4 (4)
C6—C2—S1	120.5 (4)	C5—C4—H4	121.8
C3—C2—S1	114.2 (3)	C3—C4—H4	121.8
C4—C3—C2	102.1 (3)	C4—C5—H5	122.9
C4—C3—H3	128.9	C6^i^—C6—C2	178.7 (7)
C2—C3—H3	128.9		
			
C6—C2—C3—C4	179.9 (4)	C2—C3—C4—C5	0.8 (5)
S1—C2—C3—C4	−1.1 (4)		

Symmetry codes: (i) −*x*, −*y*+1, −*z*.
